# Evolutionary Dynamics and Pathogenicity Analysis of Feline Panleukopenia Virus in Xinjiang, China

**DOI:** 10.3390/microorganisms12112205

**Published:** 2024-10-31

**Authors:** Hanwen Zhang, Wenxiang Zhang, Yu Pan, Honghuan Li, Tao He, Qianqian Dong, Wenyan Song, Wenya Zhang, Liyan Zhang, Kashaf Kareem, Song Jiang, Jinliang Sheng

**Affiliations:** 1 Department of Veterinary Medicine, College of Animal Science and Technology, Shihezi University, Shihezi 832000, China; aa747582693@163.com (H.Z.); z302729814@163.com (W.Z.); yupanshzu@163.com (Y.P.); lhh121004@126.com (H.L.); ht960704@163.com (T.H.); 18699332880@163.com (Q.D.); songwenyan1998@163.com (W.S.); zhangwyshzu@163.com (W.Z.); 15729916090@163.com (L.Z.); kashafkareem55@gmail.com (K.K.); 2 Animal Hospital, Shihezi University, Shihezi 832000, China; 3 Department of Zoology, Government College University, Faisalabad Layyah Campus, Layyah 31200, Punjab, Pakistan

**Keywords:** feline panleukopenia virus, evolutionary dynamics, VP2 protein mutation

## Abstract

Feline panleukopenia virus (FPV), a globally pervasive and highly pathogenic pathogen, has garnered significant attention recently due to the cross-species transmission of its variants. Despite the vast body of research conducted on FPV, studies exploring its evolutionary history, dynamics, and the factors driving its evolution remain scarce. The pathogenicity of strains with the prevalent mutations (A91S and I101T) in the VP2 protein has also not been fully elucidated. This study conducted a comparative analysis of FPV VP2 sequences sourced from Xinjiang province in China, other provinces in China, and other countries. It was confirmed that the evolutionary rate of FPV approached that of RNA viruses, at approximately 1.13 × 10^−4^ substitutions/site/year. The study reconstructed molecular models of the VP2 protein with the A91S and I101T mutations and used viral strains carrying these mutations to perform the animal regression experiment. It was confirmed that isolates with the A91S and I101T mutations could cause typical leukopenia and acute enteritis symptoms, suggesting that the mutant strains still possess certain pathogenicity. This is the first study to report on the evolutionary dynamics of FPV in Xinjiang, China, and it emphasized the importance of continuously monitoring FPV evolutionary dynamics.

## 1. Introduction

Feline panleukopenia is a highly acute infectious disease caused by the feline panleukopenia virus (FPV) [1]. FPV is a single-stranded DNA virus, a member of *Carnivore protoparvovirus 1*, in the *Parvoviridae* family [2]. FPV is mainly spread through the oral-nasal route and targets rapidly dividing cells, particularly the small intestine crypt epithelium, lymphoid tissue, and bone marrow. Animals of all ages in the *Felidae*, *Mustelidae*, and *Procyonidae* family can be infected by it, with mortality rates as high as 50–80%. FPV has become one of the pathogens most severely harming felines [3]. VP2 is the main capsid protein of the virus particle, accounting for more than 80% of the total capsid protein of the virus, and is a critical factor in determining the viral tropism, host range, and pathogenicity [4]. The most common strategy to prevent FPV is vaccination with the feline viral rhinotracheitis, calicivirus, and panleukopenia (FVRCP) vaccine. This peptide vaccine was previously shown to provide immunity to all three infectious diseases without interfering with each other [5,6].

Canine parvovirus (CPV) is closely related to FPV. Since its discovery, it has undergone multiple evolutionary events, and new antigenic types have constantly emerged [7]. These novel antigenic types have rapidly disseminated, posing significant challenges to established public health protection measures. In contrast, FPV has remained devoid of novel antigenic types since its identification, and prior investigation has underscored a markedly lower evolutionary rate than CPV [8], suggesting that FPV might be characterized by a relatively stable evolutionary pattern. However, in recent years, FPV has expanded its host range. It was first isolated from monkeys in 2008 [9], and later from giant pandas in 2018 [10]. In 2019, a strain of FPV characterized by the A300P mutation was isolated and confirmed to have the ability to infect canines [4]. Furthermore, research revealed the potential zoonotic transmission risk of a giant panda-derived FPV variant, as evidenced by its ability to infect human cells in vitro [11]. On top of that, the spillover of FPV might accelerate its evolution and affect its homeostasis. Given the mounting evidence indicating potential changes in the evolutionary stability of FPV, it has become critically important to delve deeper into its evolutionary history and dynamics, as well as the factors driving its evolution.

However, existing studies tend to focus on discovering novel viral variants and analyzing them for phylogeny, with a notable lack of comprehensive analyses of the evolutionary trajectories of FPV strains across different countries and years. Additionally, the pathogenicity of the widespread A91S and I101T mutant strains also remained elusive [12]. Therefore, this study conducted a comparative analysis of FPV VP2 sequences sourced from Xinjiang province in China, other provinces in China, and other countries to affirm the evolutionary dynamics of FPV and evaluate whether positive selection and genetic recombination were the driving factors of FPV evolution. Moreover, we obtained genome-wide information on an FPV strain with the A91S and I101T mutations isolated from Xinjiang, reconstructed a molecular model of its VP2 protein, and evaluated its pathogenicity in cats. In conclusion, this study revealed the evolutionary dynamics and the pathogenicity of FPV in Xinjiang, emphasizing the necessity of continuous observation of its evolutionary dynamics.

## 2. Materials and Methods

### 2.1. Sample Collection

From April to October 2023, 160 anal swab samples of sick cats with diarrhea, enteritis, and elevated body temperature were tested in 30 pet hospitals in Urumqi and 15 pet hospitals in Shihezi, Xinjiang. Among them, 31 were FPV colloidal gold positive, and the remaining cases were caused by other viruses, bacteria, and other factors that could also cause the above clinical symptoms. All 31 identified clinical samples were used for FPV isolation experiments. Finally, 12 samples could cause the cytopathic effect after blind passage in cells, and the remaining samples could not cause the cytopathic effect. Therefore, twelve FPV strains were obtained in this study.

### 2.2. Virus Isolation and Identification

To isolate the FPV, twelve fecal samples were vortex-shaken in 20% suspensions (*w*/*v*) in DMEM. The suspension was frozen at −80 °C and thawed three times, and then centrifuged at 7000 r for 20 min at 4 °C. The supernatant was filtered through a sterile 0.22 μm pore membrane filter and added into a 1% Penicillin-Streptomycin-Amphotericin B solution. The filtrate was synchronously inoculated at the time of Crandell-Rees Feline Kidney (CRFK) cell passaging. The resulting FPV cultures were continually passaged in CRFK cells until viral propagation was stable. Viral DNA was extracted using the FastPure viral DNA/RNA Kit (Vazyme, Nanjing, China). PCR was performed using FPV-specific primers (FPV-F: 5′-TAACTCCTCTGACTCCGGAC-3′; FPV-R: 5′-ACCACCGTCTGGTTGAACTG-3′) that identify a 750-bp fragment in the FPV genome sequence (nt 2062–2819, GenBank accession no. M38246). The primers were designed using Primer Premier 5.0 software [13]. The reaction program was initiated with 95 °C pre-deformation for 5 min, followed by 94 °C deformation for 30 s, 55 °C annealing for 50 s, and 72 °C extension for 45 s for a total of 30 cycles, and a 72 °C final extension for 10 min. The indirect immunofluorescence assay used a feline panleukopenia virus monoclonal primary antibody (Anti Biological Technology Co., Shenzhen, China) and Alexa Fluor 488-labeled goat anti-mouse IgG secondary antibody (ZSGB-Bio, Beijing, China). Furthermore, the virus was negatively stained with 1% phosphotungstic acid after ultracentrifugation and sucrose density gradient centrifugation, then imaged using a Hitachi-7700 transmission electron microscope (Hitachi, Ltd., Tokyo, Japan) to observe the virus particle morphology.

### 2.3. Sanger and Next-Generation Sequencing

Mutations in the VP2 gene affect the antigenicity, hemagglutination, and host range of FPV. Consequently, VP2 is often used as a target gene in molecular epidemiologic studies of FPV [14]. To obtain information on the VP2 gene of the Xinjiang isolates, viral DNA was extracted using FastPure viral DNA/RNA Kit (Vazyme, Nanjing, China), amplified using specific primers (VP2-F: 5′-AAGCCGGTGCAGGACAAGTAA-3′; VP2-R: 5′-CCAACCACCCACACCATAACAAC-3′), and the full-length VP2 gene underwent Sanger sequencing (a 2078-bp fragment; genome sequence nt 2745–4823; GenBank accession no. M38246). The primers were designed using Primer Premier 5.0 software [13]. The reaction program was initiated at 95 °C pre-deformation for 5 min, followed by 94 °C deformation for 30 s, 60 °C annealing for 2 min, and 72 °C extension for one minute and 45 s for a total of 30 cycles, then a 72 °C final extension for 10 min. By comparing the amino acid sequences, the Xinjiang isolates in this study were divided into two groups: the strains containing A91S and I101T mutations, and strains containing only the I101T mutation. We focused on the strains containing A91S and I101T mutations (there were seven strains with this feature), and the XJ-SHZ-2 strain was randomly selected from these strains for ultrafiltration concentration to obtain the complete genome information of the Xinjiang isolated strain. The viral DNA was then extracted using the methods described above. The sequence datasets containing 150-bp paired-end reads were generated by Shanghai Tanpu Biotechnology Co., Ltd. (Shanghai, China) using the Illumina NovaSeq 6000 System (Illumina, San Diego, CA, USA). Low-quality reads and contaminating sequences were removed to obtain clean reads, and the sequences were assembled by SPAdes 3.14.1 and SOAPdenovo 2.04 software. Subsequently, gene annotation was performed through the Prokka 1.14.5 companion software.

### 2.4. Phylogeny and Selection Pressure Analysis

To further analyze the genetic evolutionary features of the FPV strains isolated in Xinjiang, FPV and CPV VP2 gene sequences were randomly selected from the National Center for Biotechnology Information nucleotide database (GenBank) to align with this study’s sequences (A total of 42 sequences). CLUSTAL W was chosen as the program of alignment [15]. The Tamura 3-parameter (Gamma) model of MEGA 11.0 software [16] was used, and 1000 bootstrap replicates were performed to build the neighbor-joining (NJ) tree. The iTOL website (http://itol.embl.de/), accessed on 10 November 2023, was used to visualize the phylogenetic tree [17]. Given the divergence in objectives for subsequent analyses, the inclusion of sequence data from out clade CPV was deemed inappropriate, resulting in a discrepancy between the number of sequences utilized in subsequent analyses and those in the phylogenetic analysis. Subsequently, we assessed whether the FPV was under positive selection pressure and whether there were potential positive selection sites. After aligning this study’s sequences with the reference sequences (A total of 95 sequences, Table S1), we compared the one-ratio model with the free-ratio model using the branch model configurations in the CODEML program of PAML 4.7 software [18] to evaluate the FPV nonsynonymous rate (d_N_) to synonymous rate (d_S_) ratio ω (ω = d_N_/d_S_). Purifying selection was determined when 0 < ω < 1, while ω = 1 indicated neutral selection and ω > 1 suggested positive selection. Two model pairs, M1a (neutral) vs. M2a (selection) and M7 (beta) vs. M8 (beta and ω) were run to screen for sites subject to positive selection in the FPV VP2 gene. The likelihood ratio test was applied to assess the best model in the nested model. The significance of the above two model pairs was tested by the chi-squared (χ^2^) distribution between the 2ΔlnL values and the degrees of freedom relationship. The model was accepted when it passed the likelihood ratio test (LRT) with *p* < 0.05. Subsequently, the positive selection sites were detected by applying the Bayes empirical Bayes method, where sites were considered positively selected if the posterior probability (PP) value was greater than 0.9. Additionally, positive selection sites were verified using the FUBAR (Fast, Unconstrained Bayesian AppRoximation), FEL (Fixed Effects Likelihood), SLAC (Single-Likelihood Ancestor Counting), and MEME (Mixed Effects Model of Evolution) programs of the HyPhy 2.5 software [19].

### 2.5. Gene Recombination and Divergence Time Analysis

The sequences of gene recombination analysis and divergence time analysis were consistent with those of selection pressure analysis (A total of 95 sequences, Table S1). Gene recombination analysis was performed using the Chimaera [20], MaxChi [21], BootScan [22], RDP [22], GENECONV [23] and SISCAN [24] programs in RDP 4.0 software. We used BEAST 1.10.4 software [25] to analysis the divergence time. The best model combinations were determined using the following methods: marginal likelihood estimates of all combinations were tested in BEAUti using the path sampling/stepstone sampling method, and the one with the largest value was considered to be the best model combination [26]. Finally, the GTR + I + G4 model was selected in the BEAUti program as the nucleotide substitution model [27], and the Coalescent Bayesian Skyline was applied under strict clock models. The Markov Chain Monte Carlo (MCMC) algorithm was run 400 million times and logged every 40,000 states. Tracer 1.7.2 software checked the convergence of all parameters. The data were only considered valid if all the effective sample size (ESS) values were >200. TreeAnnotator 1.10.4 software was used to discard the 10% aging samples and obtain the maximum clade credibility (MCC) tree from the posterior tree distribution. FigTree 1.4.4 software generated the phylogenetic tree with the time scale, 95% highest probability density (HPD), and PP values. The iTOL website was used to color-code the continents where the virus originated [17].

### 2.6. Phylogeoraphic Analysis and Molecular Modeling of VP2 Protein Mutations

The sequences of phylogeographic analysis were also consistent with those of selection pressure analysis (a total of 95 sequences, Table S1). In conducting discrete phylogeographic analysis, the BEAUti program was used to add the isolated strain’s location information as traits and select a symmetric substitution model as the discrete trait substitution model. Other settings were consistent with the divergence time analysis. Subsequently, SpreaD3 performed Bayes factor (BF) calculations, assessing whether statistically significant virus migration paths exist between countries. To visualize the effect of amino acid mutations on the VP2 protein of the XJ-SHZ-2 strain, we chose a template from the Protein Data Bank (ID: 1FPV) to reconstruct a 3D structural model of the FPV VP2 protein, and utilized the mutagenic protein function of PyMOL 2.3 software, an open-source molecular visualization system, to simulate the post-mutation conformation [28].

### 2.7. Pathogenicity Assesment of FPV XJ-SHZ-2 Strain in Cats

#### 2.7.1. Animal Regression Experiments

Eight Nulla luctus felis (4 males, 4 females) aged ten weeks and weighing 1.3 to 1.5 kg were purchased from a pet market (Urumqi, China). Colloidal gold testing confirmed that all cats were free of feline panleukopenia virus, feline calicivirus, and feline viral rhinotracheitis. Two female and two male cats were randomly selected as infected (*n* = 4) and control (*n* = 4) groups, respectively. Prior to conducting the animal experiment, we embarked on a two-week disinfection process to guarantee the absence of environmental pathogens. Additionally, both groups of cats were acclimated in the new room for a week before beginning the experiment, thereby mitigating the potential stress reaction associated with the novel environment. The infected group received 1 mL orally and 1 mL subcutaneous injection of 10^4.25^ TCID50/0.1 mL XJ-SHZ-2 strain per animal. The control group received PBS in the same modes and volumes, and was kept separate from the infected group. The viral titer and dose were based on the report of Fei-fei and co-workers [29]. The groups were observed daily for defecation, mental status, ocular and nasal secretions, and appetite. Additionally, the body weight and rectal temperature of the cats were measured.

#### 2.7.2. White Blood, Neutrophil and Lymphocyte Cell Counts

Whole blood was collected every two days to continuously monitor white blood, neutrophil, and lymphocyte cell counts using the Veterinary Hematology Analyzer BC-30 Vet (Mindray Animal, Shenzhen, China).

#### 2.7.3. Histopathology

Tissue samples were obtained from the heart, liver, spleen, lungs, kidneys, brain, duodenum, jejunum, ileum, and colon of cats who died from virus infection, and were fixed in 10% formalin at room temperature for 48 h. Subsequently, sections were prepared, stained with hematoxylin and eosin (HE), and observed using an optical microscope. The control group, which was healthy and free of abnormalities throughout the experiment, was euthanized with intravenous sodium pentobarbital (100 mg/kg), and tissue samples were processed in the same way.

#### 2.7.4. Quantitative Real-Time PCR (qPCR) for Viral Load Detection

Equal quantities (1 g) of mixed tissue samples were homogenized in 5 mL PBS and centrifuged at 7000 r for 20 min at 4 °C. Viral DNA was extracted from the supernatant and amplified by qPCR using specific primers (FPV-qPCR-F: 5′-CATTGGGCTTACCACCATTT-3′ FPV-qPCR-R: CCAACCTCAGCTGGTCTCAT) and the SYBR Green I method in a LightCycler 96 instrument (Roche Life Science, Basel, Switzerland). The primers were designed using Primer Premier 5.0 software [13]. The reaction program was initiated at 95 °C pre-deformation for 30 s, followed by 95 °C deformation for 5 s, 60 °C annealing for 15 s, and 72 °C extension for 10 s for a total of 40 cycles. The melt curve followed the instrument’s default program. The FPV viral DNA load in the tissue samples was calculated by comparing the load with that of standard plasmid viral DNA (the standard curve). The standard plasmid construction process was as follows: qPCR experiments were performed using the viral DNA extracted for next-generation sequencing in Section 2.3. The qPCR products were purified by electrophoresis on 1.5% agarose gel and identified by sequencing. The recovered gel product was ligated with pMD19-T (Takara Bio Inc., Kusatsu, Japan) and transformed into E. coli DH5α. The constructed plasmids were sent to San-gon Biotech, Co., Ltd. (Shanghai, China) for sequencing and stored at −20 °C. The assay was repeated three times for each sample, and quantitative data analysis was performed using LightCycler 96 SW 1.1 software.

#### 2.7.5. Statistical Analysis

No data were excluded from this study. One-way ANOVA was used for quantitative analysis, and two-way ANOVA and multiple comparisons model in GraphPad Prism 9.0 (GraphPad Software Inc., San Diego, CA, USA) was used for statistical analysis in other analyses, and the differences were considered statistically significant with a *p* < 0.05.

### 2.8. Ethics Statement

Cats were housed in polystyrene cages with stainless steel wire lids, and given water and food ad libitum. The room temperature was maintained at 25–26 °C with a 12 h light-dark cycle. The Ethical Committee for Animal Experiments of Shihezi University (Xinjiang, China) approved the experiments (Approval Number A2023-229). The euthanasia procedure was performed according to the laboratory animal management regulations of China.

## 3. Results

### 3.1. Virus Isolation and Identification

**Figure 1:**
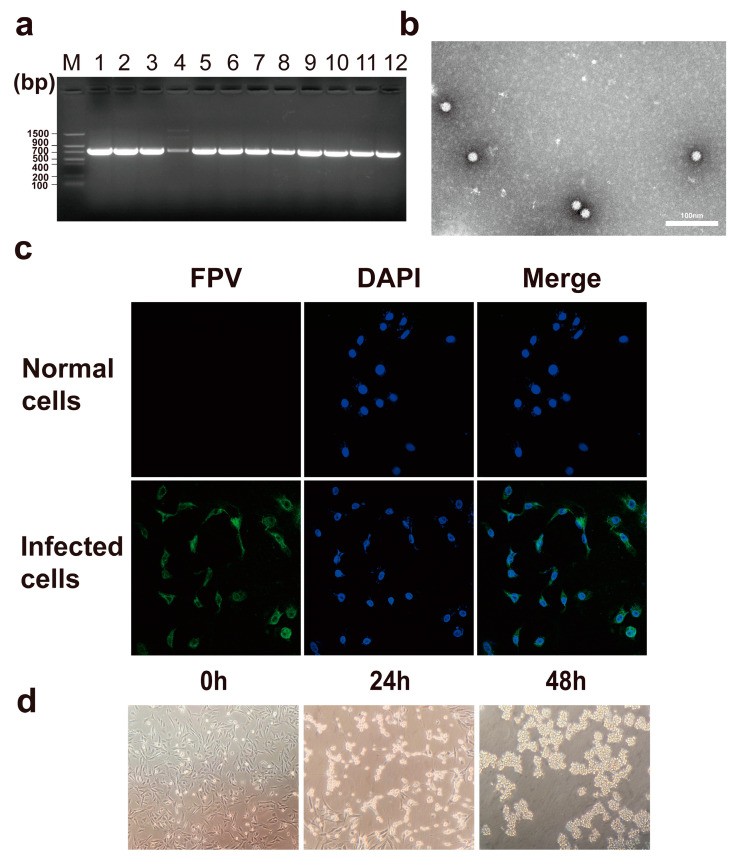
Identification of isolated FPV strains through PCR, TEM, and IFA. (**a**) PCR amplification of DNA from 12 virus isolates using specific primers and products analysis by 1.5% agarose gel. The leftmost lane is Trans DNA Marker II, and lanes 1–12 are the 12 FPV isolates. (**b**) Transmission electron microscope image after 1% phosphotungstic acid negative staining (scale bar = 100 nm). (**c**) Indirect immunofluorescence assay; normal and infected cells in the top and bottom rows, respectively. (**d**) The cytopathic effect, observed every 24 h after inoculating CRFK cells with the virus.

The PCR with the extracted viral DNA showed that all twelve isolates had a clear, bright band at 750 bp, suggesting that all were positive (Figure 1a). The viral particles, observed by transmission electron microscopy, were about 23 nm in diameter, spherical, without envelope wrapping, and with complete morphology (Figure 1b). Under fluorescence microscopy, cells infected with FPV were stained with specific green fluorescence, primarily in the cytoplasm (Figure 1c). Virus stable inheritance followed by cell inoculation can cause an apparent cytopathic effect. Over 24 h, the cells shifted from a flat fusiform shape to a converging round shape, with an increased gap between them. After 48 h, all cells in the monolayer fell off (Figure 1d).

### 3.2. Sanger and Next-Generation Sequencing

**Figure 2:**
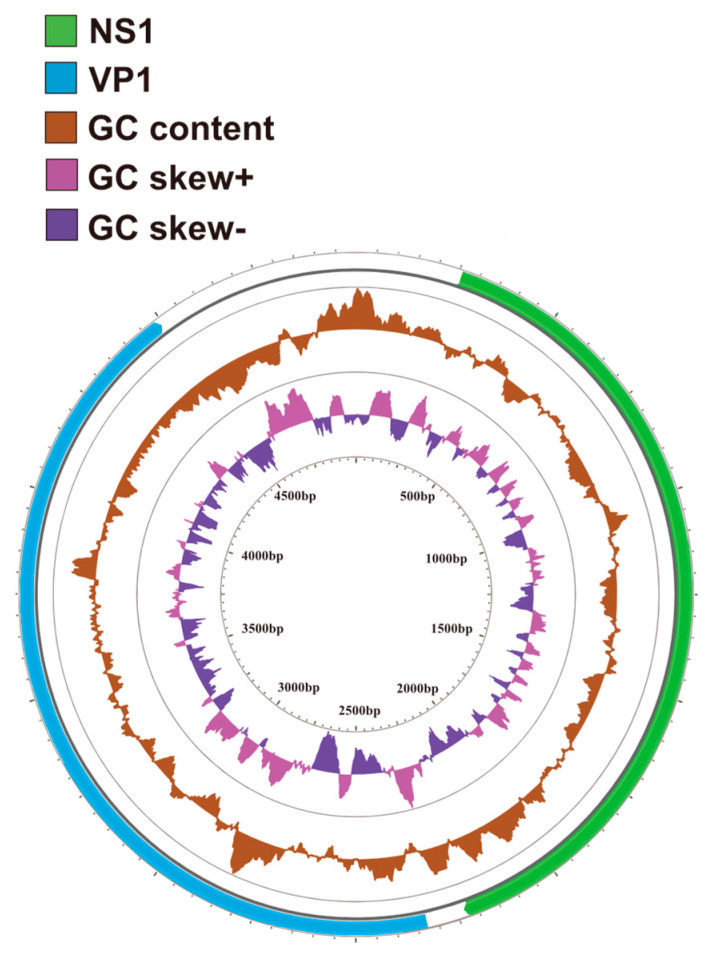
Genomic organization of the FPV; from the inside out: GC skew, GC contents, and the specific locations of the annotated CDS regions of the NS1 and VP2 genes in the genome.

The VP2 sequences from this study have been uploaded to NCBI (GenBank accession no. PQ212863–PQ212869). Full-length amplification of the isolated VP2 gene yielded a 1755 bp fragment that encodes 584 amino acids. Sanger sequencing showed that five of the twelve samples had an identical sequence, so eight VP2 nucleotide-unique virus strains were finally obtained (They are XJ-SHZ-1, XJ-SHZ-2, XJ-SHZ-3, XJ-SHZ-4, XJ-URC-5, XJ-URC-6, XJ-URC-7, XJ-URC-8, XJ is the abbreviation of Xinjiang province, SHZ is the abbreviation of Shihezi City, URC is the abbreviation of Urumqi City, the numbers correspond to the order in which they were sampled). Due to synonymous mutations, we obtained three virus strains with unique VP2 amino acid sequences after translating all the nucleotide sequences into amino acid sequences and removing duplicated ones. The nucleotide and amino acid sequences of the eight strains were compared to the standard strain sequence (GenBank accession no. M38246) for homology analysis (Table S2). Additionally, by comparing the amino acid sequences, the Xinjiang isolates in this study were divided into two groups: the strains containing A91S and I101T mutations and strains containing only I101T mutation (Table S3). We focused on the strains containing A91S and I101T mutations. XJ-SHZ-2 has this characteristic; the whole-genome sequence of the XJ-SHZ-2 strain has been uploaded to NCBI (GenBank accession no. PQ227071). The XJ-SHZ-2 sequence includes a 5′-untranslated region (UTR), two sequential Open Reading Frames (ORFs) (ORF NS and ORF S), and a 3′-UTR. The two ORFs separately encode non-structural proteins (NS1 and NS2) and structural proteins (VP1 and VP2; Figure 2). The VP2 sequence is contained entirely within the VP1 sequence, while VP1 has an additional amino-terminal sequence. The amino termini of NS1 and NS2 overlap, and the carboxyl terminus of the NS2 protein is derived from differential mRNA splicing.

### 3.3. Phylogeny, Selection Pressure and Gene Recombination Analysis

**Figure 3:**
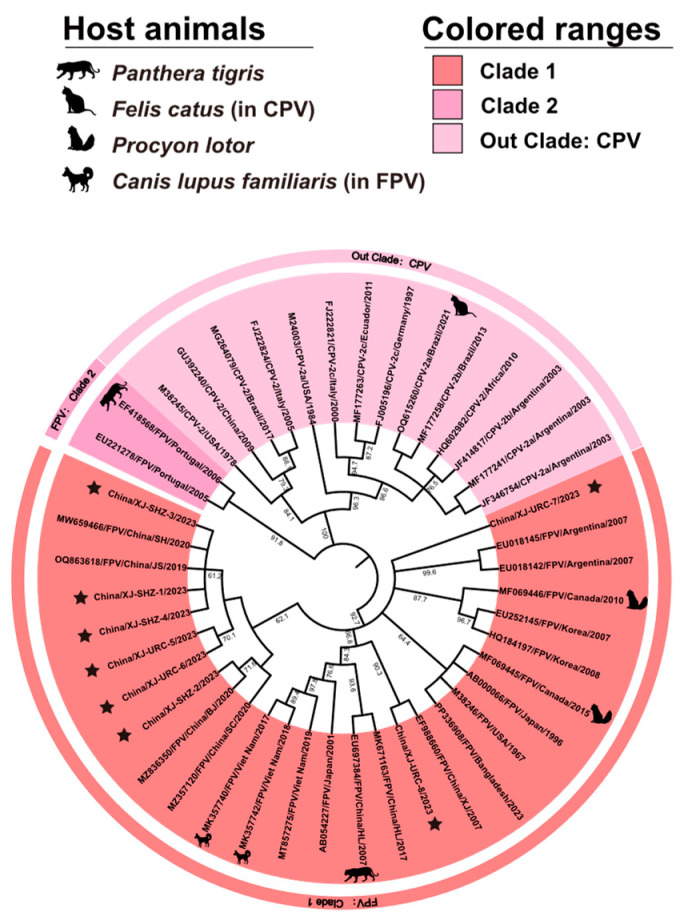
Phylogenetic analysis of FPV based on its VP2 gene sequence. CPV was used as an out group. The special FPV host animals included the *Panthera tigris* (EF418568, EU697384), *Procyon lotor* (MF069446, MF069445), and *Canis lupus familiaris* (MK357740, MK357740), and the *Felis catus* as a CPV host animal (OQ615260). These animals are presented as silhouettes. Except for them, the host animal of all sequence FPV in the figure is the *Felis catus* and CPV are all *Canis lupus familiaris*. The sequences used in this study are marked with stars.

The FPV VP2 gene NJ Tree showed two FPV clades, with all eight strains isolated in this study clustered in Clade 1 (Figure 3). The phylogenetic analysis revealed that strains from Shanghai (GenBank accession no. MW659466), Jiangsu (GenBank accession no. OQ863618), and Beijing (GenBank accession no. MZ836350), China, were closely related to strains XJ-SHZ-1, XJ-SHZ-2, XJ-SHZ-4, XJ-URC-5, and XJ-URC-6 detected in this study. The XJ-URC-8 strain was closely related to a strain from Xinjiang, China (GenBank accession no. EF988660), while the XJ-URC-7 strain was closely related to a strain from Argentina (GenBank accession no. EU018145). In the selection pressure analysis, a comparison between the one-ratio model, which assumes that all clades have the same evolutionary rate, and the free-ratio model, which assumes that each clade has an independent evolutionary rate, suggested that the one-ratio model could not be rejected since *p* = 0.99 (greater than 0.05). The one-ratio model showed that FPV had ω = 0.05 (Table 1), significantly less than 1, suggesting that the VP2 gene was subjected to a purifying selection effect and that most mutations are synonymous. A comparison between the M1a and M2a models resulted in *p* = 0.63 (greater than 0.05), indicating that the M1a model could not be rejected. Similarly, the M7 model could not be rejected because a comparison with M8 resulted in *p* = 0.057 (greater than 0.05). Therefore, the PAML 4.7 software site model detected no positive selection sites. The FUBAR model was the only model in the HyPhy 2.5 software that detected positive selection sites with a PP value greater than 0.9. These sites were at positions 91 and 322 in the amino acid sequence. Since conserved positive selection sites need to be repeatedly identified by at least two algorithms [30], it can be concluded that this study detected no positive selection sites on FPV. Notably, the selection pressure analysis used 95 virus strain sequences; 8 strains were sourced from this study, while 87 were sourced from the GenBank database. All sequences (Table S1) featured confirmed sampling times and locations. The temporal scope of these sequences spanned from 1963 to 2023, and the geographical coverage included 15 countries. These sequences were also utilized for gene recombination, divergence time, and phylogeographic analysis. To analyze whether gene recombination was the driving factor of FPV evolution, we used RDP 4.0 software for analysis but found no recombination breakpoints in any of the FPV strains.

### 3.4. Divergence Time and Phylogeoraphic Analysis

**Figure 4:**
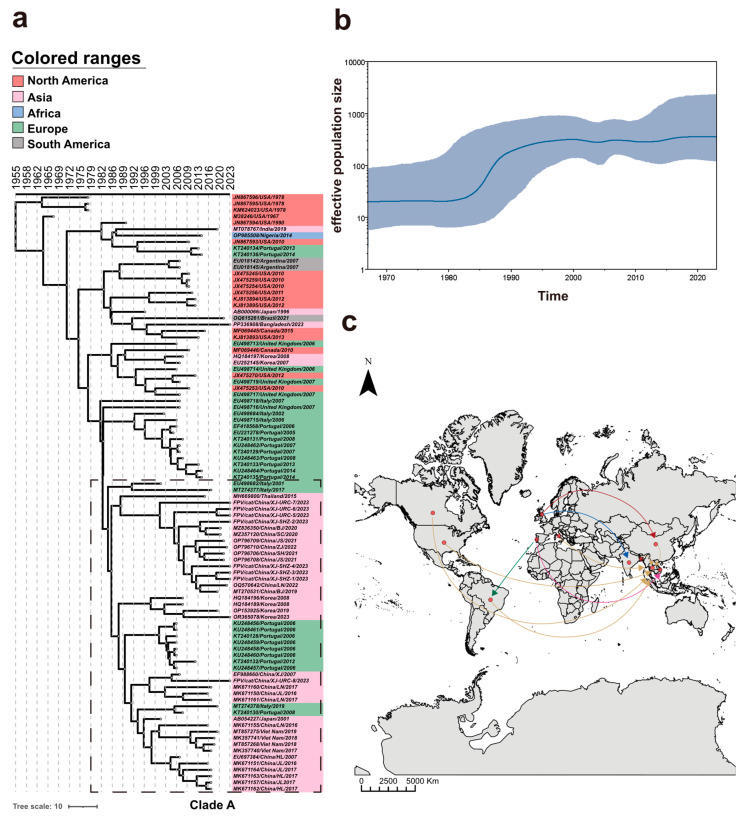
Divergence time analysis, the estimated effective population sizes over time, and discrete phylogeographic analysis. (**a**) A maximum clade credibility phylogenetic tree of FPV; the continents from which the virus originated are marked with different colors. (**b**) Population dynamics; the intervals represent 95% highest posterior densities (HPDs) of the product of generation time and effective population size. (**c**) FPV migration paths and supported migration paths (BF > 3) between countries are depicted in the figure. The route into China is indicated by the red arrow. The route into Thailand is shown by yellow arrows. The route into India is indicated by the blue arrow. The route into Vietnam is indicated by the pink arrow. The route into Brazil is indicated by the green arrow.

To analyze the evolutionary rate of FPV, the time of virus origin, and the effective population size of the virus, this study conducted a divergence time analysis and successfully constructed a MCC tree, which possesses a time scale that directly reflects the temporal evolutionary relationship among different strains (Figure 4a). The Tracer 1.7.2 software showed that ESS values of all parameters exceeded 400, indicating a satisfactory MCC tree convergence. Data analysis estimated the mean substitution rate for FPV at 1.13 × 10^−4^ (95% HPD, 8.01 × 10^−5^ to 1.48 × 10^−4^) substitutions/sites/year. The viruses analyzed in the present study were estimated to have originated in 1953.7 (95% HPD, 1940.0–1967.0). Furthermore, the estimated effective population size over time showed that the FPV population had grown slowly since the virus emerged but expanded rapidly between 1980 and 1990 and then resumed the slow growth trend (Figure 4b). Clade A (Figure 4a) was marked with a dashed box in the MCC tree. Clade A was mainly comprised of strains from Asian countries. All isolated strains obtained in this study were in this clade and were clustered with strains from other provinces in China and other Asian countries, possibly because they shared a common origin. However, some strains from European countries were also in this clade, implying that these European strains and the Asian strains were evolutionarily related, hinting at the likelihood of long-distance spatial transmission events of the virus; therefore, we then performed a phylogeographic analysis. Based on the geographic information of the various strains, we speculated a spatial transmission history of FPV across countries. The analysis was considered supported when BF was greater than 3 [31]. By this standard, ten virus migration paths (BF > 3) were found: UK to China, UK to India, Bangladesh to Thailand, USA to Thailand, Italy to Thailand, Canada to Thailand, Brazil to Thailand, Portugal to Vietnam, Portugal to Brazil, and China to Thailand (Figure 4c).

### 3.5. Molecular Modeling of VP2 Protein Mutations

**Figure 5:**
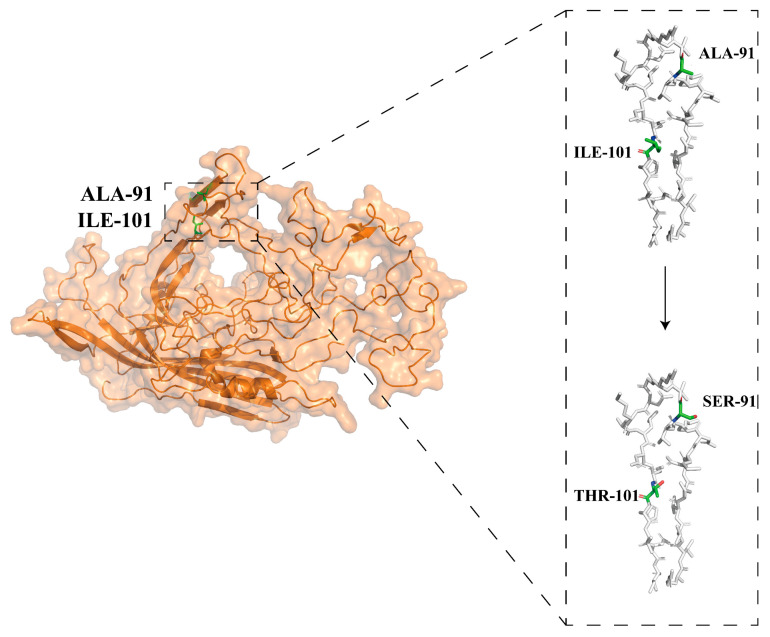
Molecular modeling of capsid protein mutations. The surface-cartoon image of the FPV capsid protein. The positions of the mutations in the XJ-SHZ-2 strain are marked as green sticks inside the dotted box. Details from the dotted box are enlarged on the right.

Molecular modelling analysis found that the FPV VP2 protein comprises eight inverted parallel β folds groups and four loops embedded in the β folds. XJ-SHZ-2 had mutations 91 (Ala to Ser) and 101 (Ile to Thr) on the VP2 protein compared with standard strains. The conformational change after A91S and I101T was shown by mutating the model protein in PyMol 2.3 software. The 91-position amino acid was on loop 1, and the 101-position amino acid was on the extended strand (Figure 5).

### 3.6. Pathogenicity Assessment of FPV XJ-SHZ-2 Strain in Cats

#### 3.6.1. Clinical Signs

**Figure 6:**
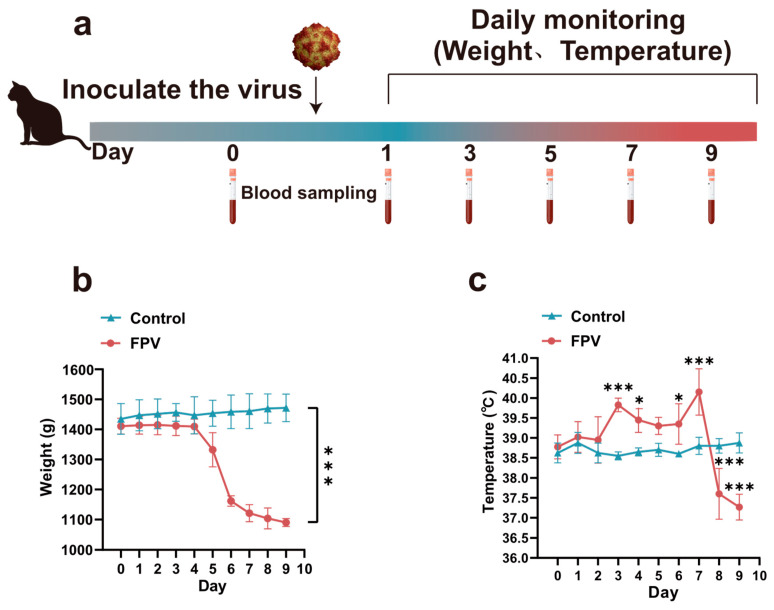
Pathogenicity assessment of the FPV XJ-SHZ-2 strain in cats. (**a**) Flow chart of the animal experiment. Blood was collected at day 0 (a day before inoculated animal), and on 1, 3, 5, 7, and 9 dpi. The body weight and temperature were measured daily. (**b**) Weight dynamics after FPV inoculation. (**c**) Temperature dynamics after FPV inoculation. Note: *** *p* < 0.001. * *p* < 0.05.

The infection period was set to 10 days. Cats in the infected group began showing symptoms of loss of appetite and lethargy at 2 days post inoculation (dpi). Their average body temperature increased to 39.8 °C (the normal range for cats is 38.5–39.5 °C) at 3 dpi, then returned to within the normal range at 4 dpi. Their body temperature suddenly rose to 40.2 °C at 7 dpi, followed by a sharp drop (Figure 6c). The average weight in this group decreased significantly at 5 dpi (Figure 6b), and the cats showed symptoms such as lethargy, increased eye and nose secretions, and watery feces. One cat in the infected group died from the viral infection at 8 dpi, and the other three cats died at 9 dpi. The cats in the control group remained healthy and showed no abnormalities throughout the entire period. The flow chart of the animal experiment is shown in Figure 6a.

#### 3.6.2. White Blood Cell, Neutrophil, and Lymphocyte Cell Counts and Tissue Viral Load Measurements

**Figure 7:**
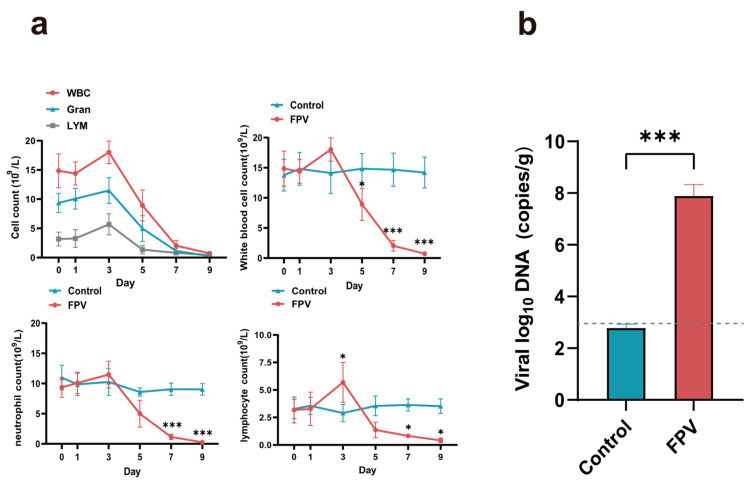
Pathogenicity assessment of the FPV XJ-SHZ-2 strain in cats. (**a**) Dynamics of the white blood cell (WBC), neutrophil (Gran), and lymphocyte (LYM) counts after FPV inoculation. The first graph shows the dynamic changes of white blood cells, neutrophils, and lymphocytes in the infected group. The following three graphs are shown for control data, visually responding to the difference in cell counts between the infected and control groups. (**b**) Virus load in the infected and control groups at 9 dpi. The limit of detection was 1 × 10^3^ copies/g marked with a dotted line. Note: *** *p* < 0.001. * *p* < 0.05.

The white blood cell, neutrophil, and lymphocyte cell counts showed an upward trend at 3 dpi, but not exceeding their respective upper normal limit values. All cell counts decreased significantly at 5 dpi, and their values approached zero at 7–9 dpi (Figure 7a). The standard curve formula was y = −3.5568x + 39.368 [R^2^ = 0.9939; x = log value of the number of copies, y = cycle threshold (Ct) value]. Quantitative PCR showed that the viral load of the infected group reached about 1.52 × 10^8^ copies/g at 9 dpi, while that of the control group was below the detection limit (Figure 7b).

#### 3.6.3. Histopathology

**Figure 8:**
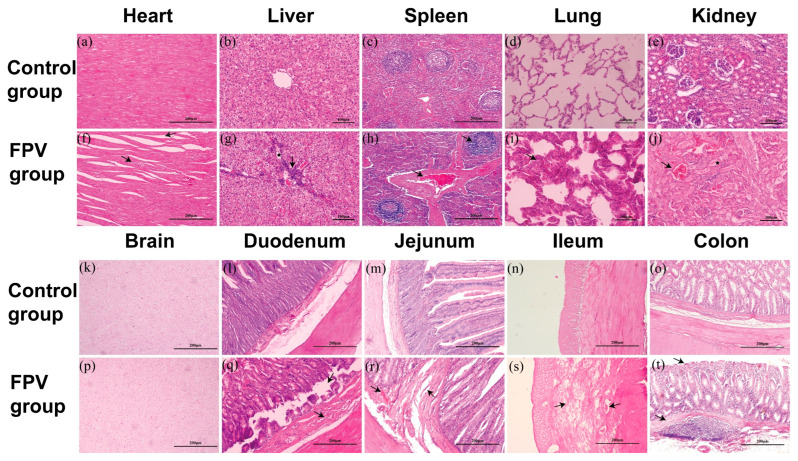
Tissue histopathology. (**a**) Heart: no abnormalities. (**b**) Liver: no abnormalities. (**c**) Spleen: no abnormalities. (**d**) Lung: no abnormalities. (**e**) Kidney: no abnormalities. (**f**) Heart: cardiac congestion and increased myocardial inter-fiber space (arrows). (**g**) Liver: hepatocellular edema (star) and inflammatory cell infiltration (arrow). (**h**) Spleen: congested blood vessels between the splenic trabeculae, and increased number of lymphocytes in the spleen corpuscle (arrows). (**i**) Lung: widened alveolar septa, dilated alveolar wall vessels, and hyperemia (arrows). (**j**) Kidney: congested glomeruli (arrow) and protein tube formation in renal tubules (star). (**k**) Brain: no abnormalities. (**l**) Duodenum: no abnormalities. (**m**) Jejunum: no abnormalities. (**n**) Ileum: no abnormalities. (**o**) Colon: no abnormalities. (**p**) Brain: no change. (**q**) Duodenum: detached mucosal layer and congested submucosal blood vessels (arrows). (**r**) Jejunum: submucosal connective tissue injury and submucosal hemorrhage (arrows). (**s**) Ileum: intestinal muscularis edema and congestion (arrows). (**t**) Colon: colonic villi abscission and intestinal muscularis edema (arrows).

Histopathological observations revealed significant abnormalities in several tissues, including myocardial congestion and an increase in the inter-fiber space, inflammatory cell infiltration and hepatocyte edema in the liver, spleen congestion and lymphocytosis, interstitial pneumonia symptoms in the lungs, kidney congestion, and protein tube formation in the renal tubules. The intestine showed edema, mucosal layer shedding, bleeding, and other symptoms. However, the brain tissues in the infection and control groups were similar. In summary, most tissues other than brain tissue show significant abnormalities after FPV infection (Figure 8).

## 4. Discussion

Cross-species transmission events of FPV have attracted much attention in recent years, as they may pose new challenges to the existing public health system’s effectiveness. Therefore, there is an urgent need to elucidate the current evolutionary pattern of FPV. This study isolated and characterized eight FPV strains from Xinjiang, China, obtaining their sequences by Sanger and next-generation sequencing. Using the sequences of 95 virus strains from 15 countries from 1963 to 2023, we analyzed the selection pressure, gene recombination, divergence time, and phylogeography to explore the evolutionary pattern of FPV in Xinjiang. Animal regression experiments assessed the pathogenicity of the strain with the non-synonymous mutations A91S and I101T. Our divergence time analysis suggested that the current evolutionary rate of FPV approaches RNA viruses [32], indicating that it has a relatively strong potential for genetic variation. The phylogeographic analysis documented the history of virus intercountry transmissions, suggesting a correlation in the genetic evolution of strains among countries. Strain XJ-SHZ-2, which had non-synonymous mutations A91S and I101T, showed pathogenicity in cats. Cats infected with the XJ-SHZ-2 strain began to show typical leukopenia symptoms at 5 days post-infection. In addition, the pathological examination showed that all segments of intestinal tissue were seriously injured, showing the characteristics of acute enteritis.

Although positive selection sites for FPV have not been detected, mutations in A91S and I101T are common in recent reports [12,33]. Based on previous studies, we have attempted to summarize the potential effects of these mutations. The FPV VP2 protein has antigen sites A and B; positions 426, 222, 224, and 93 in site A and 299, 300, and 302 in site B are critical for FPV [34]. It has been shown that changes in position 300 of the VP2 protein affect FPV binding to the transferrin receptor (TfR), which in turn affected FPV ability to infect cells [35]. Positions 91 and 101 are not among these critical sites, so we then tried to infer the possible role of these positions from the spatial structure of the protein. The tertiary structure of VP2 is mainly composed of loops 1–4 and a flexible loop [34]. Loops 1, 2, and 4 constitute the top part of the three-fold spike, while loop 3 constitutes its shoulder. The flexible loop determines the host specificity and hemagglutination activity of FPV [36]. Loop 1 is very close in space to the flexible loop, so a change of amino acids in loop 1 could affect the spatial conformation of the flexible loop and, consequently, the antigenicity, host specificity, and hemagglutination activity of FPV. Position 91 is in loop 1, so the A91S mutation might have such effects on FPV. Although position 101 is not on a loop, it is very close to loop 1, so the I101T mutation might somewhat affect the function of loop 1. Alternatively, Stucker and co-workers found that position 101 on the CPV VP2 protein affected CPV binding to the TfR [37]. Although this effect has not been verified in FPV, due to the close relationship between FPV and CPV, I101T might also affect FPV binding to the TfR.

The phylogeographic analysis detected a clear history of virus transmission from the Americas and Europe to Asia. These events might be related to the pet trade, population movements, pet food imports, and commercial trade between countries. Despite differences in methods, some of our findings concur with those of Tucciarone and co-workers [38], particularly in the FPV evolutionary rate—2.35 × 10^−4^ substitutions/site/year in their study and 1.13 × 10^−4^ in ours. However, the result obtained by Hoelzer and co-workers in their 2008 study for the FPV evolutionary rate was 8.2 × 10^−5^ substitutions/site/year [8]. This evolutionary rate was significantly lower than both our results and Tucciarone’s, suggesting that the FPV’s evolutionary rate between 2008 and 2023 has accelerated, possibly due to human activities. For example, the number of pet owners in China has increased rapidly since 2008, and the pet market has continuously expanded, presenting an advantageous environment for the dissemination and potential evolution of certain feline infectious diseases. Collectively, the available evidence indicates that FPV maintains a relatively conservative evolutionary pattern, with neither positive selection nor genetic recombination observed in this or previous research [12]. Nevertheless, the accelerated evolutionary rate and expanding host range are of concern, raising questions about whether FPV might evolve widely prevalent new antigenic types and whether new viral variants with cross-host transmission ability could lead to pandemics in the new host’s population. Future studies will necessitate sustained observations of FPV evolutionary dynamics to answer these questions comprehensively.

This study revealed changes in FPV evolution patterns, reviewed its evolution process, and highlighted important directions for future FPV research. However, due to the relatively large individual differences in cats, the initial data on body weight and body temperature in the animal experimental part of this study were slightly biased. In addition, due to practical constraints, this study could not comprehensively review the effects mutations A91S and I101T have on the virus. The XJ-SHZ-2 strain identified in this study carries both mutations and proved that it also showed pathogenicity in cats. However, pathogenicity assessment is a macroscopic analysis that ignores the microscopic details. It remains to be confirmed whether the mutation at position 91 affects the antigenicity, host-specificity, and hemagglutination activity of FPV since it is on loop 1. It is also unknown whether the mutation at position 101 affects FPV binding to the TfR as it does for CPV. Our future research will aim to verify these conjectures through experiments.

## 5. Conclusions

The evolution and genetic characteristics of feline panleukopenia virus strains in Xinjiang, China, were analyzed. The results showed that the evolutionary rate of the feline panleukopenia virus was close to that of RNA viruses, and it has accelerated significantly compared to 2008. Animal regression experiments showed that the isolated strain with A91S and I101T mutations also showed pathogenicity in cats. The effects of A91S and I101T on the pathogenic mechanisms of the virus and the functions of the viral VP2 protein need to be further studied.

## Figures and Tables

**Table 1:** The result for branch model (PAML) tests of genes.

Gene	Model	d_N_	d_S_	ω (d_N_/d_S_)	−LnL
*VP2*	One-ratio model	0.04	0.81	0.05	4023.68

## Data Availability

The original contributions presented in the study are included in the article/Supplementary Material, further inquiries can be directed to the corresponding authors.

## Supplementary Materials

The following supporting information can be downloaded at: https://www.mdpi.com/article/10.3390/microorganisms12112205/s1, Table S1: The sequences for selection pressure, gene recombination, divergence time, and Phylogeographic analysis; Table S2: Sequence identities between the strains of this study and the standard strain; Table S3: Amino acid residue characteristics.
