# Supplementary material for: Evolutionary Dynamics and Pathogenicity Analysis of Feline Panleukopenia Virus in Xinjiang, China
Source: Microorganisms. 2024 Oct 31;12(11):2205. doi: 10.3390/microorganisms12112205 (PMC11596581; doi:10.3390/microorganisms12112205)
Supplement: Supplementary file 1 [file microorganisms-12-02205-s001.zip › microorganisms-3254848-supplementary.pdf]

Supplementary Materials

Table S1. The sequences for selection pressure, gene recombination, divergence time, and Phylogeographic analysis.

	Year	Country
Reference sequences		
M38246	1967	USA
JN867595	1978	USA
JN867596	1978	USA
KM624023	1978	USA
JN867594	1990	USA
JN867593	2010	USA
JX475245	2010	USA
JX475259	2010	USA
JX475254	2010	USA
JX475256	2011	USA
KJ813894	2012	USA
KJ813895	2012	USA
JX475270	2012	USA
JX475253	2012	USA
KJ813893	2013	USA
MF069446	2010	Canada
MF069445	2015	Canada
MT078767	2019	India
AB000066	1996	Japan
AB054227	2001	Japan
PP336908	2023	Bangladesh
EU252145	2007	Korea
HQ184197	2008	Korea
HQ184196	2008	Korea
HQ184189	2008	Korea
OP153925	2019	Korea
OR365078	2023	Korea
MH669800	2015	Thailand
EU697384	2007	China
EF988660	2007	China
MK671150	2016	China
MK671151	2016	China
MK671155	2016	China
MK671164	2017	China
MK671163	2017	China
MK671157	2017	China
MK671162	2017	China
MK671160	2017	China
MK671161	2017	China
MT270531	2019	China
MZ836350	2020	China
MZ357120	2020	China
OP796709	2021	China
OP796708	2021	China
OP796706	2021	China
OQ570642	2022	China
OP796710	2022	China

MK357740	2017	Vietnam
MT857268	2018	Vietnam
MK357741	2018	Vietnam
MT857275	2019	Vietnam
EU221278	2005	Portugal
EU418568	2006	Portugal
KU248456	2006	Portugal
KT240128	2006	Portugal
KU248459	2006	Portugal
KU248458	2006	Portugal
KU248457	2006	Portugal
KU248460	2006	Portugal
KT240129	2007	Portugal
KT240130	2008	Portugal
KT240132	2012	Portugal
KU248461	2006	Portugal
KU248462	2007	Portugal
KU248463	2008	Portugal
KT240131	2008	Portugal
KT240133	2013	Portugal
KT240134	2013	Portugal
KT240136	2014	Portugal
KU248464	2014	Portugal
KT240135	2014	Portugal
EU498713	2006	United Kingdom
EU498714	2006	United Kingdom
EU498719	2007	United Kingdom
EU498717	2007	United Kingdom
EU498716	2007	United Kingdom
EU498684	2002	Italy
EU498682	2001	Italy
EU498715	2006	Italy
EU498718	2007	Italy
MT274377	2017	Italy
MT274378	2019	Italy
EU018142	2007	Argentina
EU018145	2007	Argentina
OQ615261	2021	Brazil
OP985508	2014	Nigeria
This study		
XJ-SHZ-1(PQ212863)	2023	China
XJ-SHZ-2(PQ227071)	2023	China
XJ-SHZ-3(PQ212864)	2023	China
XJ-SHZ-4(PQ212865)	2023	China
XJ-URC-5(PQ212866)	2023	China
XJ-URC-6(PQ212867)	2023	China
XJ-URC-7(PQ212868)	2023	China
XJ-URC-8(PQ212869)	2023	China

Table S2. Sequence identities between the strains of this study and the standard strain.

	Nucleotide Identity (%)	Amino Acid Identity (%)
Reference Strains		
M38246(USA,1967)	100	100
This Study Strains		
XJ-SHZ-1/2023	99.5	99.7
XJ-SHZ-2/2023	99.5	99.7
XJ-SHZ-3/2023	99.5	99.5
XJ-SHZ-4/2023	99.4	99.7
XJ-URC-5/2023	99.5	99.7
XJ-URC-6/2023	99.5	99.7
XJ-URC-7/2023	99.5	99.7
XJ-URC-8/2023	99.5	99.8

Table S3. Amino acid residue characteristics.

	Amino Acid Residues	
Reference Strains	**91**	**101**
M38246(USA,1967)	A	I
This Study Strains		
XJ-SHZ-1/2023	S	T
XJ-SHZ-2/2023	S	T
XJ-SHZ-3/2023	S	T
XJ-SHZ-4/2023	S	T
XJ-URC-5/2023	S	T
XJ-URC-6/2023	S	T
XJ-URC-7/2023	S	T
XJ-URC-8/2023	A	T
